# Female and male-controlled livestock holdings impact pastoralist food security and women’s dietary diversity

**DOI:** 10.1186/s42522-020-00032-5

**Published:** 2021-01-25

**Authors:** H. Gitungwa, C. R. Gustafson, E. Y. Jimenez, E. W. Peterson, M. Mwanzalila, A. Makweta, E. Komba, R. R. Kazwala, J. A. K. Mazet, E. VanWormer

**Affiliations:** 1 Department of Agricultural Economics, University of Nebraska-Lincoln, Lincoln, NE USA; 2 Health for Animals and Livelihood Improvement (HALI) Project, Iringa, Tanzania; 3 Departments of Pediatrics and Internal Medicine and College of Population Health, University of New Mexico Health Sciences Center, Albuquerque, NM USA; 4 Department of Veterinary Medicine and Public Health, Sokoine University of Agriculture, Morogoro, Tanzania; 5 One Health Institute and Karen C. Drayer Wildlife Health Center, School of Veterinary Medicine, University of California, Davis, CA USA; 6 School of Veterinary Medicine and Biomedical Sciences, School of Natural Resources, University of Nebraska, Lincoln, NE USA

**Keywords:** Food security, Dietary diversity, Pastoralists, Tanzania, Gender, Resource control

## Abstract

**Background:**

Food insecurity is a global problem that requires a One Health approach. As many households in low- and middle-income nations rely on crops and livestock that they produce to meet their household’s needs, food security and nutrition are closely linked to the health of animals and the environment. Resources controlled by women are more often allocated to uses that benefit the entire household, such as food, health, and educating children, than men’s resources. However, studies of gender control of resources among pastoralist societies are scant. We examined the effect of female and male control of livestock resources on food security and women’s dietary diversity among households from one agro-pastoralist and two pastoralist tribes in Iringa Region in south-central Tanzania.

**Methods:**

We conducted surveys with 196 households, which included questions on food availability and food consumption among women, livestock holdings, gender control of livestock and livestock product income, and household demographics, as well as open-ended questions on the use of income. Food availability and food consumption responses were used to construct food security and women’s dietary diversity indexes, respectively. We conducted mixed effects logistic regression to analyze how household food security and dietary diversity were associated with livestock and other household variables. We also examined qualitative responses for use of income controlled by women and how the household obtained income when needed.

**Results:**

Female-controlled livestock generally supported better household nutrition outcomes. Greater chicken holdings increased the probability of being food secure in pastoralist households but decreased it in agro-pastoralist households, while increasing the probability of having medium-high dietary diversity among all tribes. Male-controlled livestock holdings were not related to food security status. Women used income to supplement food supplies and livestock they controlled as a primary response to unanticipated household needs.

**Conclusions:**

Our results show that female-control of livestock is significantly related to household food security and dietary diversity in pastoralists and agro-pastoralists in rural Tanzania. Importantly, the relationship between food security and dietary diversity differs among tribes for both male and female-controlled livestock, which suggests that blanket policies regarding management of livestock holdings may have unintended consequences.

**Supplementary Information:**

The online version contains supplementary material available at 10.1186/s42522-020-00032-5.

## Background

The Food and Agriculture Organization reports that the recent trend of slowly falling food insecurity appears to have stalled over the last 2 years, leaving around 822 million food insecure people, the same as in 2010 [17]. The etiology of food insecurity is complex and should be considered using a One Health approach. In low- and middle-income countries, many households predominantly rely on locally produced or even home-grown food [55], inextricably connecting their well-being to the health of their animals and their environment. For instance, decreased water availability and eroded soils will reduce crop yields and animal forage, which can lower the household’s food availability and wealth.

Food and the environment are also indirectly linked through the responsibilities that women have in many households. Women are often responsible for food production, which frequently includes a major role in agricultural tasks, and for other important needs of the household, such as fetching water, gathering firewood, tending to animals, and food storage and preparation [30, 33, 34]. Due to social norms, women tend to have less authority over the use of household resources than men, despite the fact that women tend to use resources in ways that benefit the entire household [46].

While food insecurity is a consequence of poverty, it can also contribute to or prolong bouts of poverty [4, 13, 50]. Food insecurity has potential long-term consequences, particularly when it interferes with optimal growth and cognitive development in early childhood. Physical effects of food insecurity and consequent poor diet quality, including stunting and wasting, are common among children in many low- and middle-income countries [17] and pose a significant problem in sub-Saharan Africa [1]. Inadequate nutrition, including the mother’s nutritional status during pregnancy, also influences cognitive development, which can have negative effects later in life on important outcomes, such as educational attainment and livelihoods [2, 5, 32]. Food insecurity has also been linked to poor mental health in adults, which may reinforce a household’s status as food insecure [24, 25, 31].

While a household’s resources, including household assets, savings, and natural resources, such as land and water, critically influence food availability, intra-household distribution of resources, including the distribution of food among household members, also plays a key role in determining each person’s nutritional status. Over the last few decades, many have examined the impact of gender on household decision making and resource allocation to various uses, including food and nutrition, health and health-related products, and education [7, 8, 16, 28, 35, 49, 53]. The gender of the individual who earns, controls, and spends money has important implications for household outcomes, since resource control by women tends to increase household spending on food, health, and education, bringing benefits to all household members [28, 46, 53]. This finding is particularly robust in low- and middle-income countries, where cultural norms may explain these differences. In many cultures, women are expected to possess “maternal altruism,” which refers to the devotion of a woman’s energies and earnings to their families’ well-being, especially the wellbeing of her children [58]. Findings from research on gender and resource control have implications for the design of development programs that provide direct aid to households. These results suggest that an increase in male income does not improve household educational and nutritional status as much as an increase in female income would [15, 36, 52], which partially explains why empowering women has become a consistent goal in international development projects.

Pastoralists present an interesting and important case of the relationship between resource control and food security because they tend to have highly conservative gender roles and cultural norms that strongly influence community members’ behaviors [29], and their food security is closely linked to the health and productivity of their livestock. Pastoralists’ livelihoods and nutritional outcomes have traditionally been based in animal husbandry, raising domestic animals like cattle, goats, sheep, chickens, and camels, which provide them with food products, such as milk, meat, and blood, as well as wealth and cultural value [18, 51]. However, there is significant concern about the sustainability of traditional pastoralist ways of life in the face of reduced availability of natural resources, e.g., food and water, due to climate change and restrictions on movement to traditional grazing lands due to increasing settled, and frequently agricultural, populations [27, 54]. Many pastoralist communities have been abandoning or reducing traditional movements among different pasture locations and adopting a more sedentary existence [20], which allows them to practice agriculture.

Since their livelihoods, well-being, and household wealth rely heavily on domestic animal herding, traditional male and female responsibilities tend to govern pastoralists’ internal household economy [29]. In many pastoralist societies, livestock is a gendered asset [56]. In East Africa, pastoralist women frequently raise and sell poultry, but do not have control over larger, more valuable livestock and are kept from working in the labor market outside of their household [29, 56]. The acceptance of female control over poultry production and sale of poultry and poultry products has led to the adoption of poultry promotion projects to encourage female empowerment and household well-being [21, 22, 38]. Research has demonstrated that interventions, such as poultry vaccination campaigns, can help increase poultry flock size and increase consumption of poultry products by women and children (e.g. Knueppel et al. [38], de Bruyn et al. [14]). However, there is a gap in evidence about the relationship between male and female control of resources and nutritional outcomes, such as food security and dietary diversity, in pastoralist communities. In this article, we explore the relationship between male and female resource control in the form of livestock ownership and measures of food security and dietary diversity among pastoralist and agro-pastoralist communities.

## Methods

### Study area and population

**Fig. 1:**
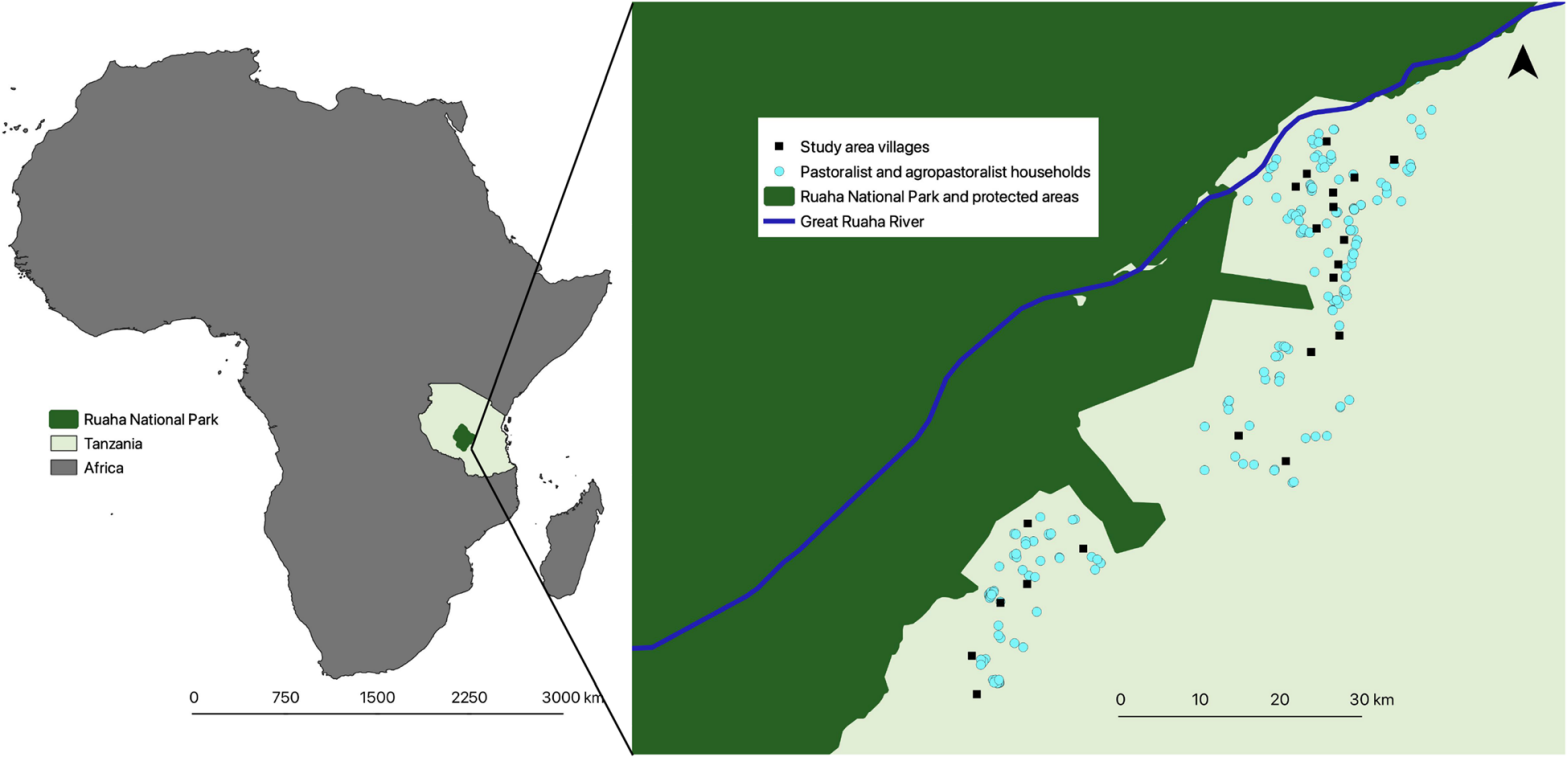
Location of study households and villages in relation to Ruaha National Park and protected areas and the Great Ruaha River in Tanzania

To assess the relationship of male and female pastoralists’ resource control with household food security and dietary diversity, we use data from a cross-sectional survey of 196 pastoralist households conducted in 2012–2013 in 21 rural villages located in Pawaga and Idodi divisions in Iringa Rural District, Iringa Region, Tanzania (Fig. 1). These divisions are located within the Rift Valley and are bordered by protected areas, including Ruaha National Park and community wildlife management areas.

Villages in Pawaga and Idodi divisions are primarily populated by Hehe and Bena agriculturalists, while pastoralist and agro-pastoralist livestock-keepers from three predominant ethnic groups, the Barabaig, Maasai, and Sukuma, typically inhabit marginal lands outlying the village houses and farms. The Maasai and Barabaig have traditionally been “pure” pastoralists, nomadic or semi-nomadic groups that move seasonally to access pasture and rely on their livestock. The Sukuma, on the other hand, are agro-pastoralists, maintaining large herds of animals while also practicing agriculture for home consumption and marketing. In many areas of East Africa, factors including economic opportunities, access to social services, and land use changes (e.g. agricultural expansion and gazetting of lands for protected areas) have reduced pastoralists’ movements [20]. The Maasai and Barabaig have become more sedentary in response to these constraints and have diversified their livelihoods. Similar to the Sukuma, the majority of the Maasai and Barabaig in the study area have established permanent households to raise crops annually and to send at least one child to school.

In the study area, significant environmental changes related to a changing climate, human resource use, and upstream development projects have decreased the availability of resources to pastoralist and agro-pastoralist communities. The Great Ruaha River, an important source of water for humans, animals, and agriculture in the study area, as well as an important source of hydroelectric power for the nation, ceased flowing for a period during the dry season in 1993 and has been dry for part of the year every year since [57]. Much of dry-season agriculture and livestock production in the study area is dependent on wetlands associated with the river [44].

#### Household surveys

Our survey consisted of three modules: a household-level livestock health and economics module, a household-level food security module, and an individual woman-level dietary diversity module. The livestock health and economic module included questions about the number of livestock owned, number of wives, family size, annual income from large animals, chickens, and livestock products, annual income from crop sales, land ownership, annual income from other sources, head of the household characteristics (age, sex, and education), wives’ education, number of wage earners in the household, whether the household received remittances, and tribal affiliation. Households were asked open-ended questions related to income controlled by women, such as money earned from the sale of poultry or eggs. Households that reported having female-controlled income were asked about their use of that income. Households were also asked what they did when they had an unexpected important expense that required them to obtain funds quickly. The open-ended questions did not include any prompts to avoid inducing respondents to provide what they might have perceived to be the researcher’s preferred answer. Questions in the livestock health and economics survey were adapted from USAID Demographic and Health Surveys (DHS) as well as previously implemented local livestock health and livelihood surveys [23, 42].

The food security module was adapted from the Household Food Insecurity Access Scale [HFIAS] [12] and Months of Adequate Household Food Provisioning [MAHFP] instruments [6]. The HFIAS assesses household food insecurity over a four-week time frame, asking nine questions about the occurrence and frequency of food insecurity conditions [12]. The MAHFP estimates household food provisioning over a one-year time frame (the previous 12 months) as a proxy for household food access [6]. The women’s dietary diversity module was adapted from the Food and Agriculture Organization (FAO) of the United Nations’ guidelines for measuring household and individual dietary diversity [37]. The questionnaire assessed consumption of different food groups (starchy staples; dark green leafy vegetables; other vitamin A rich fruits and vegetables; other fruits and vegetables; organ meats; meat and fish; eggs; legumes, nuts and seeds; and milk and milk products) over 24-h and 7-day periods.

The information used to define male and female control of livestock was collected in focus groups and pilot surveys with pastoralist households in the study area [23] as well as through informal discussions with households and local informants. Information from focus group discussions and household survey responses consistently identified male control of large animals (cattle, sheep, and goats) and female control of poultry, which was corroborated by informal conversations with households and local informants.

#### Selection of participating households and survey administration

The sample was constructed for an education intervention with these pastoralist households. The data reported in this paper were collected in a baseline round before the intervention occurred. We applied a stratified random sampling technique to select households to approach about participation, with the 21 villages in Pawaga and Idodi divisions constituting the strata. To generate a census of pastoralist households in Pawaga and Idodi villages, we produced a list of households living in each village by consulting village leaders, pastoralist leaders, and other pastoralist community members as key informants. A household consisted of all people who live in the same compound, a cluster of buildings and livestock enclosures, who shared meals or living accommodations, with one head of household. After a list of pastoralist households was assembled for each village, we selected a random sample of ten households. In some cases, it was not possible to include ten households in a village because there were a limited number of pastoralist households in the area; one village only had six resident pastoralist households, while two closely adjacent villages, one of which reported only four pastoralist households, were combined for random selection of participants. A total of 196 households were enrolled in the study.

Data on food security, livestock holdings, and household characteristics were collected at the household level, while data about the diversity of foods consumed by women were collected at the individual level. The household head (or another member of the household involved in livestock production decisions, if the head of household was not available) responded to the household-level livestock health and economics module. Women responded to questions about the number of chickens owned and use of poultry products. The senior woman with decision-making authority (or another woman involved in household food preparation, if the senior woman was not available) responded to the food security module. Data collection on women’s dietary diversity was completed with women directly, to provide a more accurate view of their reported consumption. Adult female participants from households in the study area who met the following criteria were asked to participate in the dietary diversity module: age range of 18–48 years old; member of the Maasai, Barabaig, or Sukuma pastoralist tribes; from households that owned at least ten cattle, sheep or goats; available during the study period (not planning to move out of the study area for at least 2 years); and willing to accept visitors in the home. If a woman had a chronic medical condition that required frequent medical attention (≥2 health clinic visits per month), she was excluded from the study. Because the practice of polygamy is common among these three tribes, some households had more than one wife. In that case, up to three wives between 18 and 48 years of age in a household were recruited. A total of 262 adult women from the 196 households answered dietary diversity questions.

For quality assurance, we built in the ability to validate certain key variables—for example, livestock numbers and losses—by collecting data on these variables in multiple ways that should add up if the respondent answers reliably. We have also been able to examine the validity of responses by examining data gathered in subsequent years to see whether answers to questions are consistent. We additionally used consistency in responses across households for certain variables as another check of validity by, for instance, examining reported prices for livestock, livestock products, etc. at local markets. We made use of all of these data checks when assembling the final dataset used for the analysis.

Trained Tanzanian research team members collected the module data via interviews with the participants in Swahili. Surveys were translated from English to Swahili and back-translated into English to ensure that questions were interpreted as intended. The surveys were pretested with representatives from the tribal communities who lived outside of the study area. Additionally, local enumerators who were familiar with the tribal languages were able to assist if any misunderstanding with Swahili arose [23]. Open-ended responses were recorded in Swahili and translated to English prior to data analysis. Household livestock health and economics surveys were collected from November 2012 to January 2013. Household food security and women’s dietary diversity surveys were implemented from July–September 2013.

### Data processing and analysis

Tropical livestock units (TLU) were calculated using the number of reported cattle, sheep, and goats owned by the household. One TLU was equal to one cow, 10 sheep, or 10 goats [40]. HFIAS score was calculated as a continuous measure of household food insecurity ranging from 0 (lowest level of food insecurity) to 27 (highest level of food insecurity) based on reported responses [12]. Households were then categorized [12] into one of four levels of household food insecurity: food secure (household experiences none of the food insecurity (access) conditions, or just experiences worry, but rarely), mildly food insecure (household worries about not having enough food sometimes or often, and/or is unable to eat preferred foods, but only rarely), moderately food insecure (household sacrifices quality more frequently, by eating undesirable foods sometimes or often, and/or has started to cut back on quantity by reducing the size of meals or number of meals, rarely or sometimes), and severely food insecure (household has increasingly cut back on meal size or number of meals often, and/or running out of food, going to bed hungry, or going a whole day and night without eating). We created a bivariate measure of food insecurity: food secure and food insecure, which aggregated the categories, mildly, moderately and severely food insecure [19] due to data sparsity in the sub-categories of food insecure households.

MAHFP was calculated by subtracting the total number of months out of the previous 12 months that the household reported being unable to meet their food needs [6]. Average MAHFP was determined by summing the total MAHFP for all households and dividing by the number of households surveyed [6]. Percentage of households reporting being unable to meet their food needs was also calculated by month of the year and season..

To calculate women’s dietary diversity score, responses were used to assess consumption of the nine different food groups [37]. Women were then categorized into levels of dietary diversity: low dietary diversity (consumption of 3 or fewer food groups), medium dietary diversity (consumption of 4 to 5 food groups), and high dietary diversity (consumption of 6 or more food groups). Medium and high dietary diversity were combined into one category for analysis: medium-high (MH) dietary diversity because only eight women had high dietary diversity. Since the data for this study were collected, a new indicator has been recommended (Minimum Dietary Diversity – Women), but the way in which our data were collected according to the old standard precluded calculating the new MDD-W (FAO and FHI 360, 2016).

To analyze the survey data, we calculated descriptive statistics and used mixed effects logistic regression analyses conducted with R statistical software [48]. A significance level of α < 0.05 was used for all statistical tests. We selected the independent variables for inclusion in the final model by retaining biologically and statistically significant variables that improved the model fit, using the Akaike Information Criterion (AIC). Initial variables considered in the models were chosen based on previous literature; these variables included demographic variables, such as adult education, number of wives, family size, agricultural land holdings, whether a woman in the household was currently pregnant, and other sources of income, such as receiving remittances from family members. In the regression analyses, we dropped observations from households that had a missing value for one or more of the variables included in the model. First, we dropped six households that were headed by widows since the analysis examines male and female resource control. The primary source of missing data was that the head of the household was away at the time that the survey was administered and the respondent was not certain of the answer, which resulted in random missing responses. Finally, one respondent cut the interview short after growing tired of answering questions.

We used mixed effects logistic regression to examine the relationship between food security status and wealth (livestock holdings) controlled by men versus women, while controlling for other regressors. Because we only had one observation per household of the food security status variable, the random effect was included at the village level. To examine women’s dietary diversity, we used mixed effects logistic regression to examine the association between the measure of dietary diversity and male and female wealth, while controlling for other independent variables. In the dietary diversity model, we defined the random effect at the household level rather than the village level, as some households had responses from more than one woman. In the analyses, we examined interactions between tribe and livestock to allow for differences by tribe in the relationships between livestock and the dependent variables, food security and women’s dietary diversity. The interaction analysis was planned a priori based on known differences between tribes.

Finally, we used qualitative, open-ended responses collected from the household-level livestock health and economics module to broaden our understanding of the role that female resource control plays with respect to food security and dietary diversity in the study households. Respondents’ answers were coded to identify statements related to the purchase of foods or ingredients (such as cooking oil) used in food preparation. In some cases, respondents would use a general term that translates to “household needs” in response to the question. Some of these respondents provided examples of what they meant; for instance, “household needs, such as food and school fees.” Therefore, we additionally considered responses that mentioned household needs, since it was clear from their responses that many women categorized food as a household need. We separately examined responses that mentioned food specifically as the most conservative estimate of the use of female income for food, and then considered responses that either mentioned food or household needs.

## Results

### Descriptive statistics

Participant households’ characteristics are reported in Table 1. Most participant households were Maasai (61.7%), followed by Sukuma (23.0%) and Barabaig (15.3%). Approximately 95% of surveyed households planted at least one crop in the year of the survey, and many households experienced crop failures (for instance, 28% of households’ maize crops failed, 25% of bean crops failed, and nearly 58% of squash crops failed).

**Table 1:** Characteristics of the surveyed pastoralist households (*N* = 196): Iringa Rural District, Iringa Region, Tanzania

Household characteristics	Mean (SD) /% for categorical variables	N
Cattle, sheep, and goats (TLUs)^a^	52.81 (75.8)	195
Chickens	14.57 (12.3)	190
Head of household education (Any formal education = 1)	23.6%	191
Wives’ education (Any wife receiving any formal education = 1)	19.9%	196
Number of wives	1.61 (0.94)	190
Family size	13.82 (9.4)	189
Wage earners in the household (Yes = 1)	13.8%	189
Receive remittances (Yes = 1)	23.0%	196
*Ethnicity*		
Maasai	61.7%	121
Sukuma	23.0%	45
Barabaig	15.3%	30
*HFIAS score*	3.33 (6.1)	
Food secure	64.2%	122
Food insecure	35.8%	68
*WDD score*	3.52 (0.98)	262
Low dietary diversity	55.3%	145
Medium/high dietary diversity	44.7%	117

^a^*TLUs* Tropical Livestock Units. Three main species of livestock (cattle, goats, and sheep) were converted into Tropical Livestock Units (TLUs)

There were 52.8 TLU per household on average. Sukuma households owned an average of 75 TLUs, Barabaig households owned 61.5 TLUs, and Maasai households owned 42.5 TLUs. Households owned an average of 14.6 chickens. Sukuma households again had the highest holdings on average, with over 22.7 chickens per household. Barabaig households averaged 15.4 chickens, while Maasai households owned an average of 11.5 chickens. The correlation coefficient between TLU and chickens in study households was 0.008, indicating no relationship between the aggregate number of large animals (TLUs) and chickens held by households in the study.

Only 23.6% of heads of household had received any formal education, and only 19.9% of households had at least one wife who had received any formal education. About 23% of the households received remittances. Thirty-five percent of households reported some level of food insecurity on the HFIAS, with 10% of households categorized as mildly food insecure, 4% as moderately food insecure, and 21% as severely food insecure. Based on the MAHFP, between 10.6 and 13.4% of households reported being unable to meet their food needs in July–September (when the food insecurity module was conducted), whereas from January to March, between 41.5 to 62.0% of households reported being unable to meet their food needs.

Approximately 75% of women reported obtaining the majority of their food from their own (household) production while 25% obtained the majority of their food from local markets. The majority of women (55.3%) consumed three or fewer food groups (lowest dietary diversity) in the 24 h before responding to the survey, 41.2% consumed 4 to 5 food groups (medium dietary diversity), while only 3.4% consumed 6 or more food groups (high dietary diversity). Food groups most commonly consumed during the previous 24 h included starchy staples, such as maize-based foods (99.6% of women); milk and milk products (88.9%); dark green leafy vegetables (71.8%); legumes, nuts and seeds (36.2%); and meat and fish (26.6%). Fewer women reported consuming vitamin-A rich fruits and vegetables (15.3%); other fruits and vegetables (11.8%); organ meats (3.1%); and eggs (1.1%).

### Household food security

**Fig. 2:**
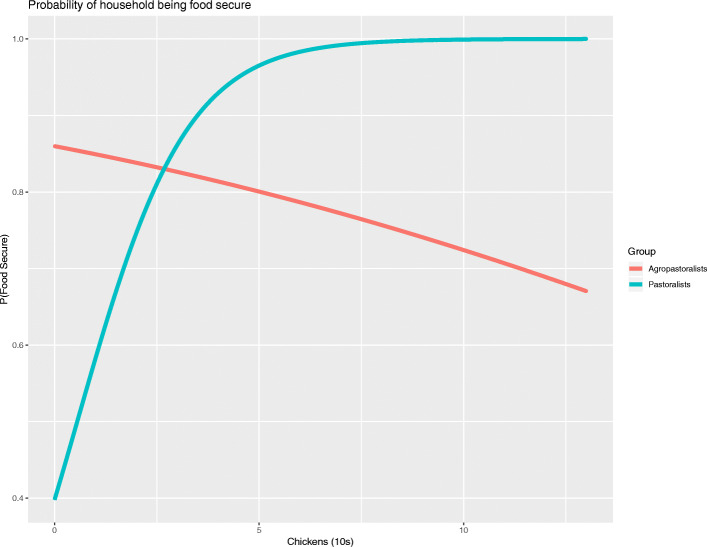
The relationship between the number of chickens (in 10s of chickens) and the probability of being food secure, by agro-pastoralist and pastoralist households

Female wealth, represented by households’ chicken holdings, was significantly associated with food security status. For pastoralist tribes, the Barabaig and Maasai, the probability of being food secure increased with the number of chickens owned by the household. Households that were agro-pastoralist, the Sukuma tribe, were more likely to be food secure than households in the pastoralist tribes that practiced less agriculture (Maasai or Barabaig) at low levels of chicken holdings. However, larger chicken flocks were associated with a lower probability of being food secure for agro-pastoralist households. Figure 2 presents the relationship between chicken flock size and food security status for agro-pastoralist and pastoralist households.

The number of wives in the household also decreased the likelihood that a household was food secure. Households with one additional wife were less likely to be food secure. Other variables included in the model were not statistically significant but improved the goodness of fit of the model (see Additional file 1: Supplementary Table 1 for relationships between individual variables and household food security). The full results of the mixed effects logistic regression of household food security are presented in Table 2.

**Table 2:** Mixed effects logistic regression of the relationship between household food security and male and female resource control, with control variables

	Odds Ratio	95% Confidence Interval
Tropical livestock units (10s)	1.03	[0.99, 1.09]
Number of wives in household	0.67	[0.44, 0.99]
Educated head of household	1.31	[0.59, 3.02]

Notes: Tribe-specific relationships between chickens and food security are presented in Fig. 2. Independent variables included in the regression were: Tropical livestock units, Number of wives in the household, Educated head of household (vs. head of household without formal education), Number of chickens owned by the household, Agro-pastoralist (vs. pastoralist) household, and the interaction between Number of chickens and Agro-pastoralist household

*N*=177; 19 households were dropped due to having data missing for at least one variable included in the regression

### Women’s dietary diversity

**Fig. 3:**
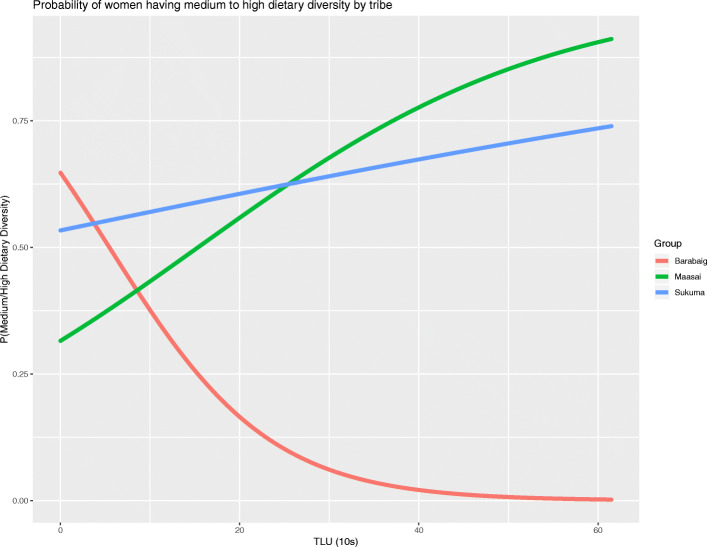
The relationship between TLU holdings (in 10s of TLUs) and the probability of a woman having medium-high dietary diversity by tribe

Chickens were associated with an increase in women’s dietary diversity. With an additional ten chickens in a household’s flock, a woman was over 1.3 times more likely to have medium-high dietary diversity. TLUs were also associated with women’s dietary diversity. However, for TLUs, the effect varied by tribe. For both Maasai and Sukuma households, greater herd size increased the probability that a woman would have medium-high dietary diversity, while for women in Barabaig households, there was a negative relationship between TLUs and the probability of having medium-high dietary diversity. The tribe-specific relationship between TLU holdings and women’s dietary diversity is presented in Fig. 3.

The number of household members and the number of wives in the household also had a statistically significant relationship with women’s dietary diversity. Each additional household member made a woman 0.94 times as likely to have medium to high dietary diversity. On the other hand, each additional wife increased the odds of a woman having medium to high dietary diversity by approximately 1.5 times. Other variables included in the model were not statistically significant but improved the goodness of fit of the model (see Additional file 1: Supplementary Table 2 for relationships between individual variables and dietary diversity). Table 3 presents the full results of the mixed effects logistic regression of women’s dietary diversity.

**Table 3:** Mixed effects logistic regression of the relationship between women’s dietary diversity and male and female resource control, with control variables

	Odds Ratio	95% Confidence Interval
Chickens (10s)	1.32	[1.00, 1.78]
Number of household members	0.94	[0.90, 0.98]
Number of wives in household	1.54	[1.08, 2.23]
Educated head of household	1.85	[0.98, 3.50]

Notes: Tribe-specific relationships between TLUs and WDD are presented in Fig. 3. Independent variables included in this regression were: Number of chickens, Number of household members, Number of wives in the household, Educated head of household (vs. head of household without formal education), Tropical livestock units, Household tribe, and interactions between TLUs and Household tribe

*N* = 241; 22 women were dropped due to having data missing for at least one variable included in the regression

### Qualitative responses on women’s use of income

Of the households reporting income from poultry products, 94% said that the income was solely controlled by women, while another 5% said that both women and men jointly made decisions about the use of those funds. One household said the man controlled the income. Out of 189 households answering questions about income, 87 households (46%) reported uses of female-controlled income in the previous 12 months. Table 4 presents data on the use of female income with respect to procuring food and household needs.

**Table 4:** The use of female-controlled income in purchasing food

Use of female income	Percent	Examples mentioned in surveys
Purchase food	48.3%	Vegetables, meat, onions, tomatoes, salt, sugar, cooking oil, milling grain, dough to make local donuts (mandazi), food for children.
Household needs (without explicitly mentioning food)	41.4%	Soap, school expenses for children (school fees, school clothes, and notebooks), medicine, beads, purchase livestock, things for church, medicine for livestock, materials to make cultural items, clothes, cosmetics, shoes.
Total potential households using female income to supplement food availability	89.7%	

Notes: 87 households reported how female-controlled income was used out of 189 households responding to the questions about female-controlled income

Of the households reporting female-controlled income in the 12 months prior to the survey, nearly 50% explicitly mentioned using that income to purchase different types of food or to pay for services that would increase the household’s food supply (for instance, paying to mill grain). Another 41% of households stated that women’s income was used for household needs, without specifically naming food items. However, since other households stated that they used female income “for household needs, like vegetables…” it is possible that some of these respondents used female income to purchase food as well. Other examples of items that respondents mentioned as being household needs included a number of things that would benefit the health or human capital of household members, such as soap, school expenses, and medicine, as well as investments related to pastoralism, like purchasing livestock and paying for medicine for livestock. Only 10.3% of households reporting female income did not mention food or household needs.

Households also reported what they did when they had an unexpected important expense that required them to obtain funds quickly. Over 50% of households mentioned using livestock to raise funds. The most frequently named type of livestock was chickens (18.4% of households), followed by “livestock” without specifying which type (15.3%), goats (10.5%), and cattle (8.4%). Other common responses included selling crops of various types, borrowing from other households, working outside of the home for pay, and selling milk.

## Discussion

We found consistent evidence that gender and resource control matter for household nutritional outcomes. For pastoralists, the number of chickens owned by a household increased the likelihood that a household was food secure and that a woman had medium or high dietary diversity (rather than low dietary diversity). For an additional 10 chickens owned, a household was over two times more likely to be food secure, while 10 more chickens were associated with being more than 1.3 times more likely to have medium or high dietary diversity.

Women’s responses to open-ended questions about the use of income earned by women corroborate the observed benefits to the household of women’s ownership of productive assets. While previous research reported that owning more chickens leads to higher consumption of eggs and meat from chickens [38], qualitative data from our surveys also showed that the income earned by women from the sale of animals and animal products is frequently used in ways that increase and diversify the households’ food supply. In many cases, the income is used to purchase food from the market, but some households reported using the income from chickens to pay for milling services to turn grain they had grown into meal or flour. Other responses also support the idea that income controlled by women is often used for pro-household purposes, such as school fees and medicine for household members and livestock, consistent with findings from other populations [7, 8, 16, 28, 35, 49, 53].

We found that Sukuma households were significantly more likely to be food secure than the Barabaig or Maasai at low levels of chicken holdings. The Sukuma are traditionally agro-pastoralists, who grow a wider variety of crops and employ sophisticated food preservation techniques; research involving populations living near protected areas to the west of our study area also found that the Sukuma have higher levels of food security [43]. For the Sukuma, the relationship between poultry holdings and food security differed from the pure pastoralist tribes. While for Maasai and Barabaig households having more chickens increased the likelihood of food security, for Sukuma households, having more chickens was associated with a decreased likelihood of food security. It is not clear what drives this relationship, though one possibility is that spending more time on poultry production crowds out other uses of Sukuma women’s time that would contribute more effectively to food security, such as time-consuming food gathering and preservation techniques. Sukuma households have been documented to have extensive knowledge of the local availability of wild foods [11, 26, 33], and in general women in Tanzania are responsible for the gathering and preservation of wild food products [33]. An alternative explanation of the inverse relationship between chicken holdings and food security is that Sukuma women put more effort into poultry production when the household is less food secure as a strategy to increase food availability through additional consumption of poultry products and the ability to purchase foods through income generated from the sale of poultry and poultry products.

The evidence of the effect of male-controlled livestock, measured in tropical livestock units, or TLUs, was mixed. Surprisingly, the number of TLUs owned by a household, which is widely acknowledged to be a critical component of pastoralists’ food supply, wealth, and cultural standing (e.g., Lybbert et al. [40]), was not significantly associated with household food security. Livestock ownership was associated with dietary diversity, though the effect was modest. Women in Maasai and Sukuma households had moderate increases in the probability of having medium to high dietary diversity, while women in Barabaig households actually had lower probabilities of more diverse diets with more TLUs. Recent research from other pastoralist areas facing similar constraints to pastoralist livelihoods found increased crop production at the same time that households were decreasing livestock holdings, while achieving the same level of food security on average [10]. While we found little evidence from our analyses of an impact of cattle, sheep, and goats on food security or dietary diversity, they may be critical in dealing with crises. Chickens were the most commonly mentioned type of animal used by households to respond to an unexpected, important need for funds; however, both cattle and goats were also named by nearly 20% of households. Given the difference in value of cattle, goats, and chickens, each type of animal may play an important role in responding to different types and scales of unexpected household needs.

The result that women from Barabaig households are less likely to have high dietary diversity when they have higher household livestock ownership could reflect forces of intrahousehold bargaining. Since cattle, sheep, and goats (the livestock that constitute the measure of TLUs) are male-controlled, higher TLUs may result in greater bargaining power for men over the use of household resources. Intertribal differences in household composition and responsibilities may also explain this result. Barabaig have the smallest average household size but have higher mean TLU holdings than the Maasai. Among some pastoralist tribes, women’s responsibilities include caring for calves and sick animals, milking cattle, distributing milk to the household members, and processing animal skins [29] and Karmebäck et al. [34] found that pastoralist women in Kenya take on greater herding responsibilities as the landscape becomes more fragmented. Allocating more time to herding or care of livestock would take time away from other activities, which could include activities that would increase the variety of foods available for consumption.

Tribal differences in the relationship between livestock holdings and both food security status and dietary diversity may reflect differences in intrahousehold dynamics among tribes. Women may have different responsibilities from one tribe to another that create tradeoffs between investing more of their labor in livestock production, whether in female-controlled poultry or helping with cattle, sheep, and goats, and time they spend producing food for the household. Intrahousehold allocation of resources depends on the relative contribution of income (typically) by members to the household [9], but cultural norms may influence how different household members’ contributions influence outcomes. For instance, women’s assets at the time of marriage are associated with more education for children in Bangladesh and South Africa, but in Ethiopia, it is the assets that men bring to a marriage that appear to contribute to greater investment in children’s education [47]. Cultural norms may also influence the opportunities that women have to contribute income to the household. In conservative societies, women may be forbidden from public spaces where they might interact with men, severely curtailing opportunities for female employment [3].

While there are some tribal differences, we found strong evidence that chickens, which are the main female-controlled resource in these pastoralist households, are associated with greater food security and higher levels of women’s dietary diversity, both of which are important for the health and well-being of the woman and, if the woman becomes pregnant, the developing fetus. Women’s answers to open-ended questions about the use of female-controlled income and how households deal with unexpected and important expenses suggest that women who raise chickens are in a position to supplement household food supplies if the food supply is insufficient and that chickens provide a store of wealth that can be sold off if unexpected needs for income arise. Many households specifically noted the importance of income from chickens and eggs in providing funds to purchase food or food production services, such as milling grain, for their households. Chickens were the most frequently mentioned type of livestock used by households to deal with unexpected expenses.

The study location is in an area of south-central Tanzania that has experienced significant human population growth and decreases in availability of natural resources critical for pastoralists, such as water and pasture, due to climate change and the development of large-scale upstream agricultural production schemes [41, 44, 45]. We found levels of reported food insecurity that were lower than a previous study in the area [39], though this may partially be explained by the timing of the survey, and a different sample population. The food security module was conducted from July to September, the months in which the fewest households (10–13%) reported not being unable to meet their food needs. This is markedly lower than the levels reported at other times of the year; for instance, 42–62% of households reported not having enough to eat during January to March. It is likely that the percentage of households classified as food insecure would have been higher if the survey had been conducted between January and March. Regardless, about one in three households reported food insecurity during likely the most food secure period of the year, with one in five reporting having to cope by cutting back on meal size or number of meals often, and/or running out of food, going to bed hungry, or going a whole day and night without eating. The majority of women surveyed (55%) reported low dietary diversity at the time of the survey, consistent with other studies in pastoralist populations. Again, it is likely that the proportion of women with low dietary diversity is higher during other times of the year.

Our cross-sectional data collection prevented us from observing changes over time or with variation in season. The measure of food security we used was specific to the month prior to when the questions were asked, and these questions were asked at the most food secure time of year for these households, as indicated by the MAHFP. Pastoralists are also highly dependent on rainfall, rain-fed pasture for their livestock, and, increasingly, rain-fed agriculture for food and water for the household and their livestock. To overcome this limitation, longitudinal data that capture the relationship between nutrition-related outcomes and gendered resource control within a varying climate would help elucidate the role of female-controlled resources in ensuring positive nutrition-related outcomes. Additionally, data on the use of time by different members of the household would be valuable to help better understand differences in the relationship between livestock and nutrition status outcomes observed among the tribes. These data would allow us to examine whether those households with higher livestock holdings, but lower food security or dietary diversity, are using time that other households spend on other food production activities for livestock rearing.

However, our results support the importance of female control of resources, even in marginalized and patriarchal groups. Female controlled resources were associated with better household food security and women’s dietary diversity, both of which are critical to ensuring the health of women and children. However, our results also highlight the importance of understanding household economies before prescribing a particular course of action to improve food security, alleviate poverty, or tackle other important development issues. For both food security and dietary diversity, we found different relationships between increasing numbers of livestock and the outcome of interest. While this variation may reflect an attempt by the household to compensate for, for instance, a failed bean harvest by building up the chicken flock to provide a steady supply of eggs, it could be problematic to uniformly push for women to increase flock sizes without a better understanding of what activities the additional time they would spend raising chickens would displace. Taking a One Health approach to improving the nutrition and well-being of pastoralist families requires fully understanding the linkages among enhanced poultry health and production, agricultural production in changing environments, and cultural influences on selling and consuming poultry and poultry products.

## Supplementary Information

**Additional file 1.** Supplementary tables.

## Data Availability

The datasets used and/or analyzed during the current study are available from the corresponding author on reasonable request.
