# Supplementary material for: Female and male-controlled livestock holdings impact pastoralist food security and women’s dietary diversity
Source: One Health Outlook. 2021 Jan 25;3:3. doi: 10.1186/s42522-020-00032-5 (PMC8011380; doi:10.1186/s42522-020-00032-5)
Supplement: Supplementary file 1 — Additional file 1. Supplementary tables. [file 42522_2020_32_MOESM1_ESM.docx]

**Female and male-controlled livestock holdings impact pastoralist food security and women’s dietary diversity**

Supplementary Table 1: Odds ratios and 95% confidence intervals from bivariate logistic regressions of household food security (dependent variable) and independent variables included in the full model in the manuscript.

| Regression | Independent variable | Odds ratio | 95% Confident Interval |
| --- | --- | --- | --- |
| 1 | Tropical Livestock Units (10s) | 1.03 | [0.99, 1.09] |
| 2 | Chickens (10s) | 1.35 | [1.03, 1.88] |
| 3 | Agro-pastoralist | 3.05 | [1.38, 7.50] |
| 4 | Number of wives in household | 0.94 | [0.68, 1.30] |
| 5 | Educated head of household | 1.31 | [0.64, 2.82] |

Supplementary Table 2: Odds ratios and 95% confidence intervals from bivariate logistic regressions of women’s dietary diversity score (dependent variable) and independent variables included in the full model in the manuscript.

| Regression | Independent variable | Odds ratio | 95% Confident Interval |
| --- | --- | --- | --- |
| 1 | Tropical Livestock Units (10s) | 1.01 | [0.99, 1.03] |
| 2 | Chickens (10s) | 1.32 | [1.07, 1.68] |
| 3 | Household tribe: Sukuma | 2.52 | [1.40, 4.60] |
| 4 | Household tribe: Barabaig | 1.14 | [0.57, 2.27] |
| 5 | Number of wives in household | 1.23 | [0.95, 1.59] |
| 6 | Educated head of household | 1.62 | [0.92, 2.86] |
| 7 | Number of household members | 0.99 | [0.97, 1.02] |
